# Homogeneous 2D and 3D alignment of cardiomyocyte in dilated cardiomyopathy revealed by intravital heart imaging

**DOI:** 10.1038/s41598-021-94100-z

**Published:** 2021-07-19

**Authors:** Kiyoshi Masuyama, Tomoaki Higo, Jong-Kook Lee, Ryohei Matsuura, Ian Jones, Chris Bakal, Shuichiro Higo, Sachio Morimoto, Shigeru Miyagawa, Yoshiki Sawa, Yasushi Sakata

**Affiliations:** 1 Department of Cardiovascular Medicine, Osaka University Graduate School of Medicine, 2-2 Yamadaoka, Suita, 565-0871 Japan; 2 The Institute of Cancer Research, London, Chester Beatty Laboratories, 237 Fulham Road, London, SW3 6JB UK; 3 Department of Cardiovascular Regenerative Medicine, Osaka University Graduate School of Medicine, 2-2 Yamadaoka, Suita, 565-0871 Japan; 4 Department of Cardiovascular Surgery, Osaka University Graduate School of Medicine, 2-2 Yamadaoka, Suita, 565-0871 Japan; 5 Department of Medical Therapeutics for Heart Failure, Osaka University Graduate School of Medicine, 2-2 Yamadaoka, Suita, 565-0871 Japan; 6 Department of Health and Medical Care, International University of Health and Welfare, Okawa, Fukuoka 831-8501 Japan

**Keywords:** Multiphoton microscopy, Cardiovascular biology, 3-D reconstruction

## Abstract

In contrast to hypertrophic cardiomyopathy, there has been reported no specific pattern of cardiomyocyte array in dilated cardiomyopathy (DCM), partially because lack of alignment assessment in a three-dimensional (3D) manner. Here we have established a novel method to evaluate cardiomyocyte alignment in 3D using intravital heart imaging and demonstrated homogeneous alignment in DCM mice. Whilst cardiomyocytes of control mice changed their alignment by every layer in 3D and position twistedly even in a single layer, termed myocyte twist, cardiomyocytes of DCM mice aligned homogeneously both in two-dimensional (2D) and in 3D and lost myocyte twist. Manipulation of cultured cardiomyocyte toward homogeneously aligned increased their contractility, suggesting that homogeneous alignment in DCM mice is due to a sort of alignment remodelling as a way to compensate cardiac dysfunction. Our findings provide the first intravital evidence of cardiomyocyte alignment and will bring new insights into understanding the mechanism of heart failure.

## Introduction

Dilated cardiomyopathy (DCM) is a non-ischemic cardiomyopathy whose prevalence is more than 1 per 250 individuals and one of the leading causes of adult heart transplantation^1,2^. Characteristics of DCM are left or biventricular dilation and systolic dysfunction in absence of coronary artery disease, hypertension, valvular disease or congenital heart disease^1^. The second leading cause is hypertrophic cardiomyopathy (HCM) which is dominantly a genetic disorder and characterized by ventricular hypertrophy, diastolic dysfunction^3^, ventricular arrhythmias^4^, left ventricular outflow obstruction, and chest pain^5^. One of the hallmarks of HCM is cardiomyocyte disarray seen in from 24.1 to 46.2%^6^ of the patients, depending on the genomic location of the mutation^7^. However, there has been reported no specific array pattern in any other aetiology of cardiomyopathy including DCM. It might be because cardiomyocyte disarray has been assessed by heart tissue with a short-axis cross section in a two-dimensional (2D) manner^8^. Recently, advance in cutting-edge microscopy and computational image processing allows us to visualise tissue structures and biological processes in a three-dimensional (3D) manner that we have never seen before in a 2D manner. Using a tissue clearing technique, we have observed 3D structure of sympathetic nerve in murine hearts with light-sheet microscopy and revealed nerve sprouting after myocardial infarction^9^.

In addition, we have recently succeeded in observing beating rat cardiomyocytes by a two-photon microscopy^10^. Multi-photon microscopy has advantages compared with conventional single-photon confocal microscopy: the excitation beam has a longer wave so that it can achieve deeper penetration and markedly reduces overall photobleaching and photodamage^11^. Intravital confocal and two-photon microscopy have been used in combination with fluorescence molecular imaging probes in a lot of research fields such as cancer research, immunology, and neuroscience^12–15^. Nevertheless, in the field of cardiology, it had been difficult to observe an intravital heart due to its dynamic movement. We have overcome this issue by developing a handmade stabilizer with gentle suctioning^10^, which enables us to capture images of myocardium and subcellular structures without photobleaching for several hours. Using this system, we intravitally observed ischemia/reperfusion injury-induced damage of cardiomyocytes and infiltration of leukocyte into a cardiac tissue^16^.

Given these backgrounds, we aimed to apply our intravital heart imaging technique for assessing 3D cardiomyocyte array as it is. Here we demonstrate that homogeneous 2D and 3D array and loss of myocyte twist in DCM model mice and that homogeneous alignment of cardiomyocytes can generate greater contractility at least in 2D.

## Results

### Establishment of 3D array evaluation using two-photon microscopy

**Figure 1:**
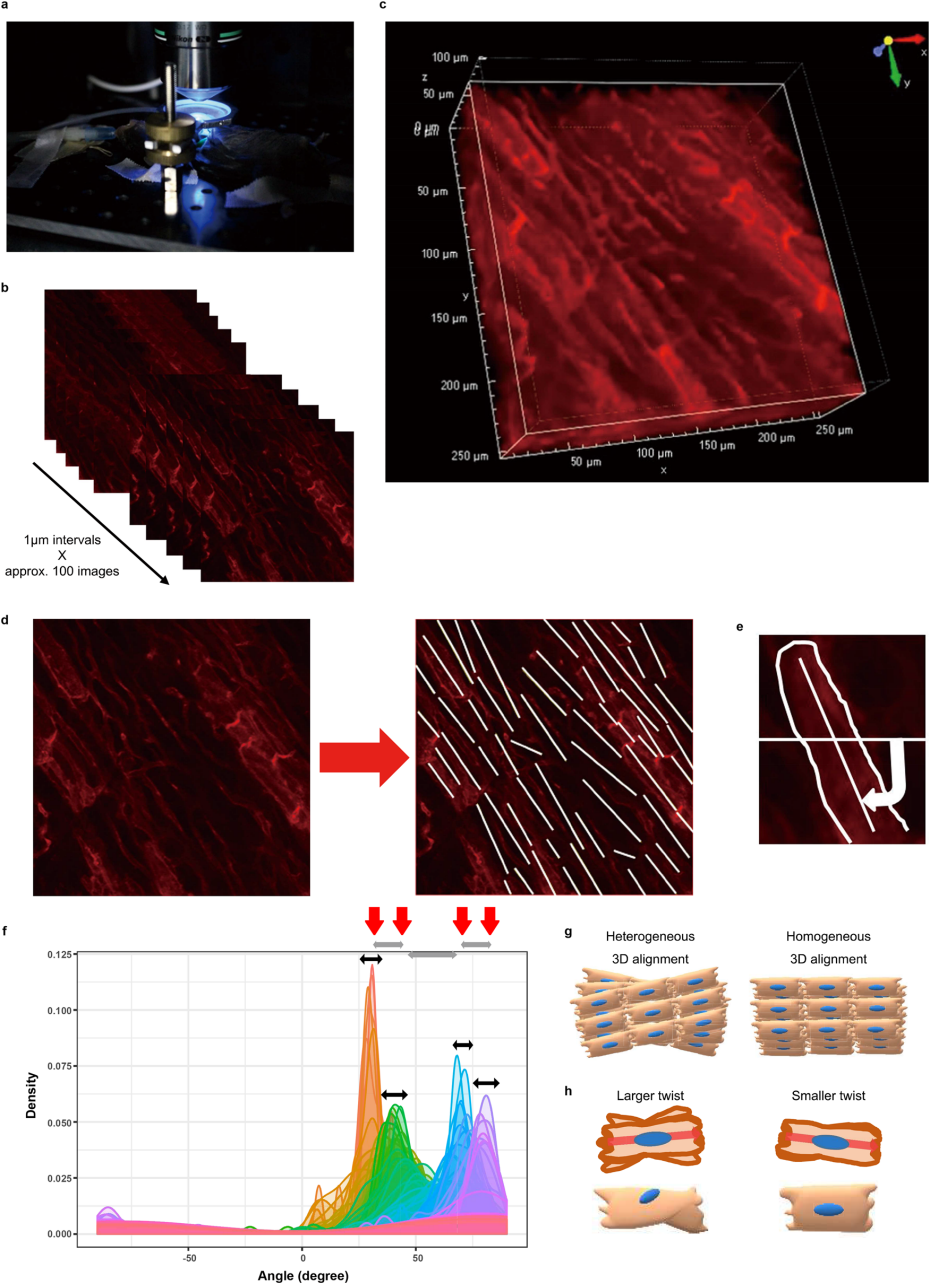
Establishment of 3D alignment evaluation using two-photon microscopy. (**a**) Experimental setup of two photon microscopy. Mice were placed on a two-axis translation stage under the objective lens of two-photon microscopy in a temperature-controlled dark box. (**b**) Sequential image stacks were captured at 1 μm intervals from surface down to approximately 100 μm depth. (**c**) 3D image reconstruction was performed using the images described in (**b**). (**d**) Longitudinal lines of cardiomyocytes were drawn manually on each layer. (**e**) Angle measurement of cardiomyocyte. Angles were measured from a horizontal line. The range of angle was from − 90° to 90°. (**f**) Angle distribution of mT/mG mice was plotted and merged layer by layer. The graph was created using the R software. Cardiomyocyte exhibited several peaks with a certain spread around each peak (black bidirectional lines), and neighbouring layers formed clusters under a single peak. Red arrows indicate medians of each cluster and grey bidirectional lines indicate distance between median values of neighbouring clusters. (**g**) Schematic images of 3D cardiomyocyte alignment. Left panel is overlapped cardiomyocytes layers with various orientation directions, representing heterogeneous 3D alignment. Conversely, right panel is those with similar orientation directions, meaning homogeneous 3D alignment. (**h**) Illustrations of cardiomyocyte twist. Upper panels are the illustrations where 2D plane images of cardiomyocytes overlapped. The left upper panel consists of 2D images with different longitudinal angles of cardiomyocytes (larger twist), whereas the right upper panel consists of those with similar longitudinal angles (smaller twist). Lower panels are the cartoons reconstructed from the upper panels.

To visualise the cardiomyocyte membrane by intravital imaging, we used global double-fluorescent *Rosa26*^*mT/mG*^ knock-in mice^17^ (mT/mG mice) as a red fluorescent reporter. Beating hearts of these reporter mice were observed using a two-photon microscopy (Fig. 1a and Supplementary Movie 1). For every session, approximately 100 sequential image stacks at 1 μm interval were obtained from the surface down to 100 μm depth and 3D image reconstruction was performed (Fig. 1b,c). On each layer, we manually drew longitudinal lines of cardiomyocytes and measured angle from a horizontal line, ranging from − 90° to 90° (Fig. 1d,e). The angle plots of all layers were merged so that layer-to-layer change of angle distribution can be visualised. In these merged plots, cardiomyocytes exhibited several peaks with a certain spread around each peak (Fig. 1f). As neighbouring layers formed clusters under a single peak, each cluster is assumed to represent an actual layer of cardiomyocytes. Also, a spread around the peak is considered to indicate the magnitude of cardiomyocyte twist within a xy plane, termed cardiomyocyte twist. To assess cardiomyocyte array quantitatively, we measured 3 parameters. First, we calculated standard deviation (SD) of the angles in every single stack image to see the alignment variation of cardiomyocytes in 2D (Fig. 1f). Large SD can be interpreted as heterogeneous 2D alignment, whereas small SD can be as homogenous 2D alignment. Second, we assessed distance between median angles of neighbouring clusters to assess the 3D placement gap between actual cardiomyocyte layers (Fig. 1f). Far distance between median angles of cluster meant heterogeneous 3D alignment, whilst short distance between median angles of cluster meant homogeneous 3D alignment (Fig. 1g). Third, we evaluated spread around peaks. The peak angle in a 2D layer image represents the direction of cardiomyocytes as a whole on that layer. Neighbouring layers with similar peak angles are supposed to project a single cardiomyocyte. Therefore, the spread around peak angles reflects longitudinal distribution of a cardiomyocyte in Z-axis direction and spread around peak angles of each cluster can be defined as “cardiomyocyte twist” (Fig. 1h). As the peak angle was almost equivalent to the median angle of each 2D layer, we calculated spread around median angles of each cluster to evaluate cardiomyocyte twist. We visibly selected the minimum and maximum median angles among neighbouring layers within a cluster and subtracted the minimum from the maximum (Fig. 1f). Wider spread represented large twist and vice versa (Fig. 1h). This method enabled us to reconstruct intravital cardiomyocyte array and quantify this not only in a 2D but also in a 3D manner.

### DCM mice demonstrated homogeneous cardiomyocyte array and less twist

**Figure 2:**
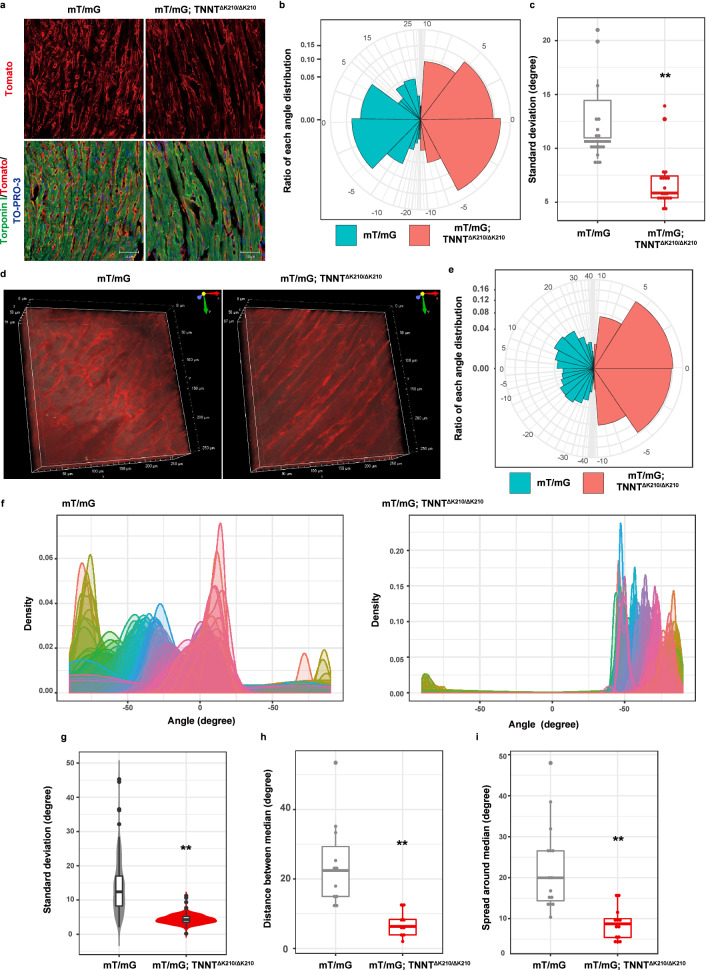
DCM mice demonstrated less various angle distribution both in 2D and in 3D. (**a**) Heart sections of mT/mG mice and mT/mG; *TNNT*^ΔK210/ΔK210^ mice were immunostained for Troponin I (green). Cardiomyocyte membranes were genetically labelled with a Tomato fluorescent reporter protein (red). Nuclei were counterstained with TOPRO3 (blue). Scale bar, 50 μm. (**b**) Angle distribution of cardiomyocytes on immunostained heart tissue sections was compared and visualised on a pie chart between mT/mG mice (light green) and mT/mG; *TNNT*^ΔK210/ΔK210^ mice (pink). On each image, longitudinal lines of cardiomyocytes were manually drawn and angles from a horizontal line was measured and calculated angles from median angle, ranging from − 90° to 90°. (**c**) Standard deviation of all the angles on each image was compared between mT/mG mice (grey) and mT/mG; *TNNT*^ΔK210/ΔK210^ mice (red, biological replicates = 4 each, 5 slices per one mouse). **P < 0.01 versus mT/mG mice. (**d**) Representative 3D views of the hearts of mT/mG mice and mT/mG; *TNNT*^ΔK210/ΔK210^ mice. Approximately 100 sequential images were recorded and reconstructed using a Nikon two-photon microscopy and its built-in software. (**e**) A pie chart showing angle distribution of cardiomyocytes on 100 images described in (**d**). (**f**) Representative layer by layer merged plots of angle distribution of mT/mG mice and mT/mG; *TNNT*^ΔK210/ΔK210^ mice. Each layer with 1 μm intervals was differently coloured. (**g-i**) Quantitative analysis of 2D and 3D cardiomyocyte alignment in mT/mG mice and mT/mG; *TNNT*^ΔK210/ΔK210^ mice. SD of the angles on every stack image (2D alignment, **g**), distance between median angles of neighbouring clusters (3D alignment, **h**), and spread around median angles of each cluster (cardiomyocyte twist, **i**) were statistically analysed between mT/mG mice and mT/mG; *TNNT*^ΔK210/ΔK210^ mice (biological replicates = 4 each). Statistical significance was determined by unpaired two-tailed Student’s t-test. **P < 0.01 versus mT/mG mice. A box and a line above/below the box indicate SD and 95% confidence interval, respectively. All statistical analyses were performed with the R software and (**b,c,e-i**) were created using the R software.

Using this quantification, we examined cardiomyocyte array of DCM model mice. We crossed mT/mG mice with mutant cardiac troponinT to generate membrane red fluorescent protein-tagged DCM model mice (mT/mG; *TNNT*^ΔK210/ΔK210^ mice). Same aged mT/mG mice were used as control mice. Consistent with previous reports, echocardiogram showed left ventricular dilatation and decreased fractional shortening in mT/mG; *TNNT*^ΔK210/ΔK210^ mice compared with mT/mG mice (Supplementary Fig. 1a, 1b, and 1c). We first analysed cardiomyocyte array with conventional immunofluorescence staining. Variation of angle distribution was smaller in mT/mG; *TNNT*^ΔK210/ΔK210^ mice than in mT/mG mice (Fig. 2a,b). SD of angles in mT/mG; *TNNT*^ΔK210/ΔK210^ mice were significantly smaller than those in mT/mG mice (Fig. 2c), suggesting 2D homogeneous alignment in mT/mG; *TNNT*^ΔK210/ΔK210^ mice.

We next compared cardiomyocyte array of mT/mG; *TNNT*^ΔK210/ΔK210^ mice with that of mT/mG mice using two-photon microscopy (Fig. 2d, Supplementary Movie 2 and 3). Variation of angle distribution was smaller in mT/mG; *TNNT*^ΔK210/ΔK210^ mice than in mT/mG mice (Fig. 2e,f). SD of angle in each layer were significantly smaller in mT/mG; *TNNT*^ΔK210/ΔK210^ mice than in mT/mG mice, which underlined homogeneous 2D alignment (Fig. 2g). Distance between median angles of neighbouring clusters were significantly shorter in mT/mG; *TNNT*^ΔK210/ΔK210^ mice than that in mT/mG mice (Fig. 2h). Spread around median angle was significantly narrower in mT/mG; *TNNT*^ΔK210/ΔK210^ mice than that in mT/mG mice (Fig. 2i). These results collectively suggest cardiomyocytes of mT/mG; *TNNT*^ΔK210/ΔK210^ mice showed homogeneous array both in 2D and in 3D and smaller myocyte twist.

**Figure 3:**
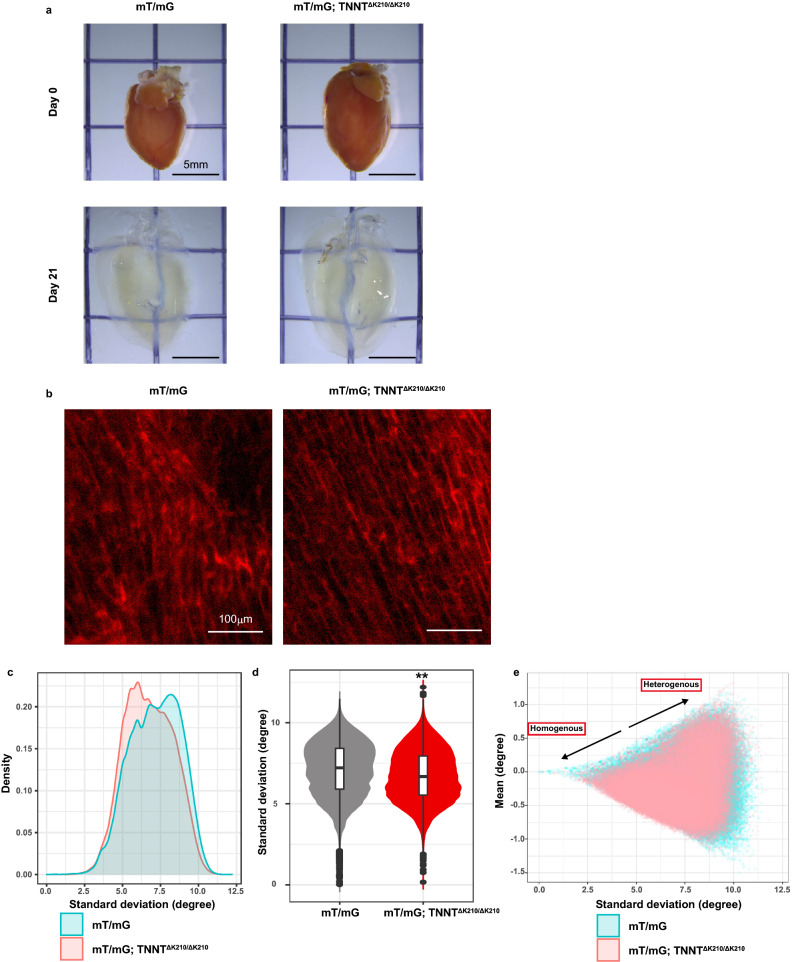
DCM mice demonstrated less various angle distribution in 3D histology. (**a**) Hearts of mT/mG mice and mT/mG; *TNNT*^ΔK210/ΔK210^ mice before (upper panels) and after (lower panels) CUBIC method. Scale bar, 5 mm. (**b**) Representative 2D image of the hearts of mT/mG mice and mT/mG; *TNNT*^ΔK210/ΔK210^ mice. Cardiomyocyte membranes were genetically labelled with a Tomato fluorescent reporter protein (red). Scale bar, 100 μm. (**c–e**) Quantitative analysis of cardiomyocyte alignment in mT/mG mice and mT/mG; *TNNT*^ΔK210/ΔK210^ mice. Distribution of Standard deviation (SD) of mT/mG mice and mT/mG; *TNNT*^ΔK210/ΔK210^ mice (**c**). SD of the orientation in cell neighbourhoods were statistically analysed between mT/mG mice and mT/mG; *TNNT*^ΔK210/ΔK210^ mice (**d**). Visualisation of all orientations in mT/mG mice and mT/mG; *TNNT*^ΔK210/ΔK210^ mice by plotting according to SD and mean orientations in neighbours. Dots located in upper right direction mean local heterogeneous alignment, whilst those located in lower left direction mean local homogeneous alignment. (**e**) Statistical significance was determined by unpaired two-tailed Student’s t-test. **P < 0.01 versus mT/mG mice. A box and a line above/below the box indicate SD and 95% confidence interval, respectively. All statistical analyses were performed with the R software and (**c–e**) were created using the R software.

To validate the intravital method, we further evaluated the cardiomyocyte array of those mice using 3D histology with tissue clearing. Hearts were transparentised and decolorized for 21 days and longitudinal images from epicardium to endocardium were obtained every 10 μm by light-sheet microscopy, where a membrane Tomato red fluorescent protein visualised cardiomyocyte borders (Fig. 3a,b). On each 2D image, the orientation of cardiomyocyte and its average and standard deviation within neighbours, which approximate alignment heterogeneity in a small region around each cardiomyocyte, were calculated using the MATLAB software. These parameters were significantly smaller in mT/mG; *TNNT*^ΔK210/ΔK210^ mice than in mT/mG mice, reinforcing homogeneous alignment in mT/mG; *TNNT*^ΔK210/ΔK210^ mice throughout the layers (Fig. 3c–e).

### Homogeneous cardiomyocyte alignment in 2D generated greater contractility

**Figure 4:**
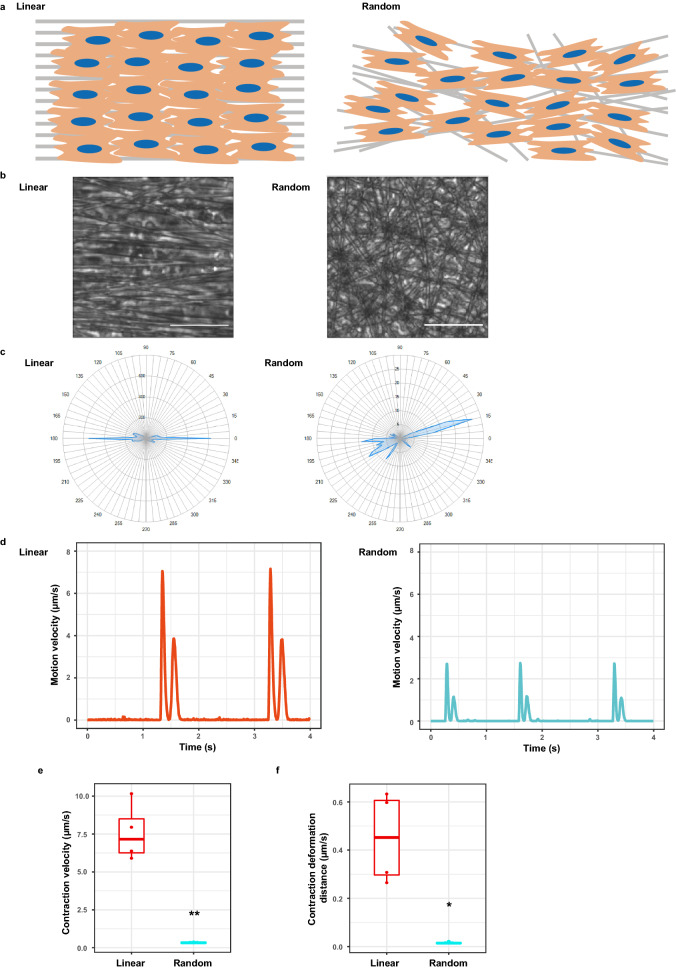
Homogeneous cardiomyocyte alignment in 2D generated greater contractility. (**a**) Cartoons of linearly and randomly aligned substrates. Neonatal rat cardiomyocytes (NRCMs) were seeded on top of them and cultured for 7 days. (**b**) Representative bright filed images of linearly and randomly aligned NRCMs. Scale bar, 50 μm. (**c**) Motion direction maps of linearly and randomly aligned NRCMs. The distribution of motion directions can be seen in these maps. (**d**) The motion velocity time-dependent curves of linearly and randomly aligned NRCMs in the 256 × 256-pixel region of interest (ROI). In each beating wave cycle, the first and second peak represented maximum contraction velocity and relaxation velocity, respectively. (**e,f**) Comparison of contraction velocity and contraction deformation distance of linearly and randomly aligned NRCMs. Contraction velocity (**e**) and contraction deformation distance (**f**) were statistically analysed (biological replicates = 4 each). Statistical significance was determined by unpaired two-tailed Student’s t-test. **P < 0.01, *P < 0.05 versus linearly aligned NRCMs. A box and a line above/below the box indicate SD and 95% confidence interval, respectively. All statistical analyses were performed with the R software and (**d–f**) were created using the R software.

We next sought to determine whether homogeneous array of cardiomyocytes plays a causative role in global cardiac dysfunction. To address this, we manipulated the alignment of cultured neonatal rat cardiomyocytes (NRCMs) and compared contractility between linearly and randomly aligned cardiomyocytes. We prepared substrates which induced NRCMs to be cultured in linear or random array (Fig. 4a) and cultured NRCMs on top of these substrates. After 7 days culture, the motion of NRCMs in the 256 × 256 pixel-region of interest (ROI) were analysed (Fig. 4b, Supplementary Movie 4 and 5). We first confirmed the direction of contraction on those different substrates. As expected, the motion on linear fibres was bidirectional, whereas that on random fibres was multidirectional (Fig. 4c). As a whole, both maximum contraction (the first peak) and relaxation velocity (the second peak) of NRCMs on linear fibres were higher than those on random fibres (Fig. 4d). We also calculated contraction velocity and contraction deformation distance and found that both of them were significantly higher in NRCMs on linear fibres compared with those on random fibres (Fig. 4e,f).

### Homogeneous 2D alignment altered contractile pattern of cardiomyocytes

**Figure 5:**
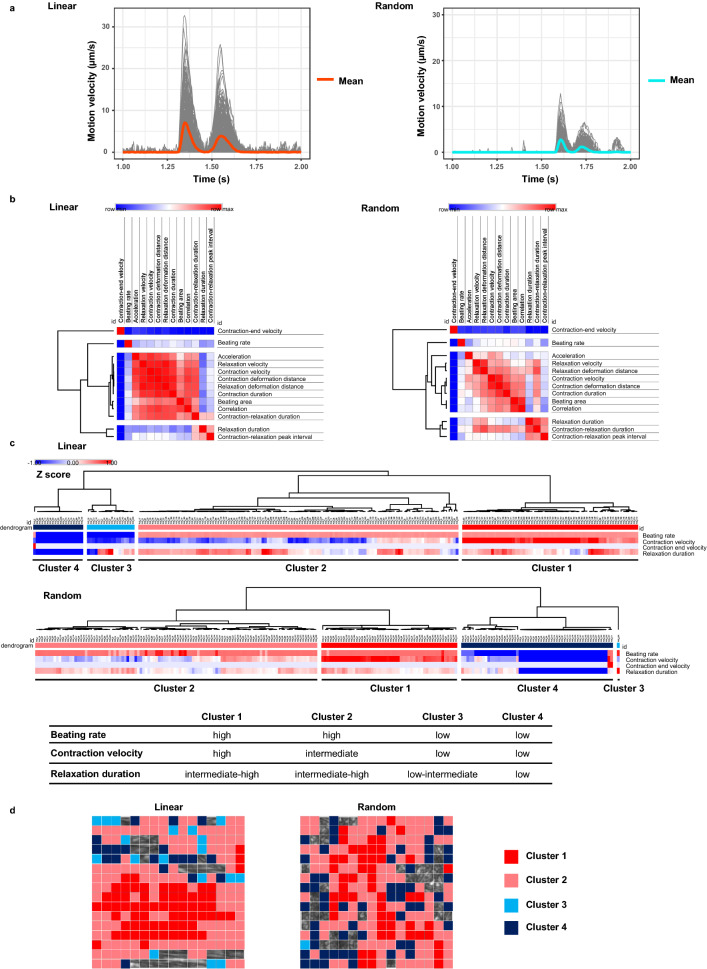
Homogeneous cardiomyocyte alignment altered contractile pattern. (**a**) Individual motion velocity time-dependent curves of 256 small ROIs (grey curves) within 256 × 256-pixel large ROIs shown in Fig. 4b. A red and light blue curve represent mean kinetics of linearly and randomly aligned NRCMs, respectively. The graphs was created using the R software. (**b**) Similarity matrices of all 13 quantitative parameters automatically extracted from individual motion velocity time-dependent curves. Correlation coefficients were calculated by Spearman’s rank correlation test. Hierarchical clustering identified 4 representative parameters: contraction-end velocity; beating rate; contraction velocity; and relaxation duration. (**c**) Hierarchical clustering of 256 small ROIs on linearly and randomly aligned NRCMs based on 4 representative parameters described in (**b**). Four distinct clusters were identified and identical across both alignment: Cluster 1: high contraction velocity, intermediate to high relaxation duration, and high beating rate; Cluster 2: intermediate contraction velocity, intermediate to high relaxation duration, and high beating rate; Cluster 3: low contraction velocity, low to intermediate relaxation duration, and low beating rate; and Cluster 4: low contraction velocity, low relaxation duration, and low beating rate. ROIs with zero values throughout the observation period were excluded from this clustering. (**d**) Large scale ROIs shown in Fig. 3b were mapped according to the 4 distinct clusters. Cluster 1, 2, 3, and 4 were coloured with red, pink, light blue, and dark blue, respectively.

To look into the mechanism by which linearly aligned NRCMs increase contractility, we split the already selected ROI into 256 small pieces of regions and analysed the motion of each region (Fig. 5a). In addition to contraction velocity and contraction deformation distance, 11 parameters were extracted from the motion velocity time-dependent curves. All those parameters were put into one similarity matrix based on Spearman’s correlation test and classified into 4 groups by hierarchical clustering (Fig. 5b). Both linearly and randomly aligned NRCMs showed similar matrices. We picked up contraction end-velocity, beating rate, contraction velocity, and relaxation duration as representative parameters for each group. Using these parameters, we then performed hierarchical clustering of 256 small regions and identified 4 distinct clusters. The features of each cluster are almost identical across linearly and randomly aligned NRCMs: Cluster 1: high contraction velocity, intermediate to high relaxation duration, and high beating rate; Cluster 2: intermediate contraction velocity, intermediate to high relaxation duration and high beating rate; Cluster 3: low contraction velocity, low to intermediate relaxation duration and low beating rate; Cluster 4: low contraction velocity, low relaxation duration and low beating rate (Fig. 5c). We finally coloured an original large ROI according to the clusters and found that Cluster 1 and 2 regions, which are considered to have greater contractility, located adjacent to each other in linearly aligned NRCMs (Fig. 5d). In contract, distribution of those clusters was relatively random in randomly aligned NRCMs. Taken together, these results suggest that homogeneous cardiomyocyte alignment reinforced contractility by converging forces into constrained regions.

## Discussion

**Figure 6:**
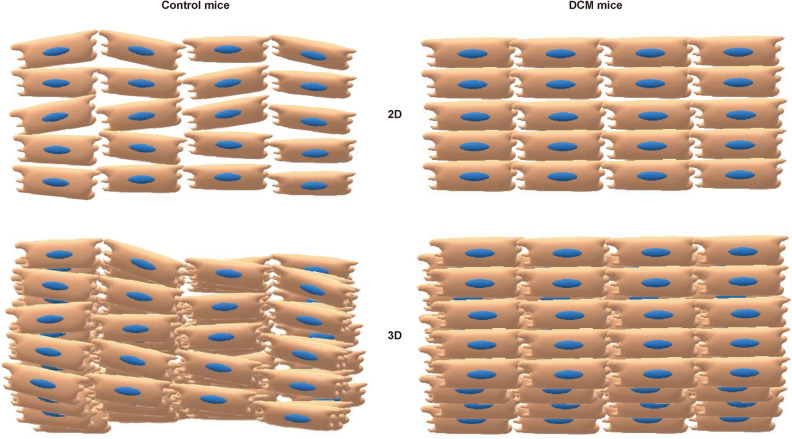
DCM mice show homogeneous 2D and 3D alignment and loss of twist. Schematic images of cardiomyocyte alignment in mT/mG (Control) mice and mT/mG; *TNNT*^ΔK210/ΔK210^ (DCM) mice. Cardiomyocytes of DCM mice exhibited homogeneous alignment both in 2D and in 3D and loss of twist compared with Control mice.

In the present study, we have established a novel method to evaluate 3D cardiomyocyte array by intravital heart imaging. Using this technique, we found that cardiomyocytes of DCM mice align homogeneously both in 2D and in 3D (Fig. 6). We also demonstrated less twist of cardiomyocytes in DCM mice, which is consistent with previous findings^18,19^. Histopathological assessment of a heart tissue taken from endomyocardial biopsy has been a powerful tool to determine the aetiology of heart failure. Indeed, approximately 30% of the patients with HCM show cardiomyocyte disarray which is an independent predictor of sudden cardiac death^4^. However, previous studies have been using cardiomyocyte histology of fixed tissues in a 2D manner^8,20^, thus lacking information of 3D array. Also, even in 2D assessment, conventional histopathological specimens only give us the information of arbitrary planes where we cannot reconstitute an in vivo structure. Although a recent study has established 3D direct measurement of cardiomyocyte volume, nuclearity, and ploidy with thick histological section^21^, this reconstitution is still far from intravital information. To overcome these limitations, we observed cardiomyocytes by an intravital heart imaging technique and reconstructed about 100 stack images so that we can visualise the cardiomyocyte array as it is. That being said, our technique highlighted the alignment only of several layers on epicardium and it cannot be excluded that the homogeneous alignment seen in DCM mice is just due to physics, i.e. that a more dilated wall with a larger ventricular diameter will result in lack of surface curvature compared to a control heart or that the DCM heart with less movement was firmly stabilised on our custom-made stabiliser. We therefore evaluated the longitudinal alignment of cardiomyocyte on myocardium (middle layer) and endocardium with conventional immunofluorescence staining and found that angle distribution in DCM mice was less variable than that in control mice on both myocardium and endocardium section, which was similar to that of epicardium (Supplementary Fig. 2). Moreover, we confirmed those findings using 3D histology with tissue clearing techniques. Light-sheet microscopic observation allowed us to visualise longitudinal cardiomyocyte alignment in a depth up to endocardium and revealed that the angle distribution in DCM mice was less variable not only on epicardium but also on endocardium. These results collectively indicate that our findings can be extended to cardiomyocyte alignment as a whole. The other limitation of this study is the frame rate of intravital imaging. It took approximately 0.53 s to capture every single image with two-photon microscopy, which means that each captured intravital image is an average of more than 2 cardiac cycles. This averaging could partly contribute to the wider angle distribution and larger mean SD of control mice in dynamic intravital imaging compared to static 3D histology, resulting in the greater SD difference between control and DCM mice in intravital imaging (Figs. 2g and 3d). Technical advances that give higher frame rates and/or image processing with electrocardiogram synchronisation would be needed to address this issue. Even with those limitations, we still believe that our technique will bring a new understanding how cardiomyocytes align and twist in vivo and how they change these in a pathological condition.

Mechanistically, why do cardiomyocytes of DCM mice align homogeneously? Is it beneficial for global cardiac function? DCM mice showed lower fractional sarcomere shortening and peak velocity of sarcomere shortening due to decreased calcium sensitivity^22^. Thus, contractility of each cardiomyocyte is supposed to be decreased. To cope with the decreased contractility of cardiomyocytes, the heart alters in size, geometry, and function, termed cardiac remodelling^23^. Cardiac remodelling is considered as an adaptive mechanism at the early stage, however, often becomes maladaptive and leads to poor prognosis when it exceeds a certain level. One of the best-known remodelling mechanisms to increase the stroke volume in DCM is cardiac enlargement^24^ which might have caused “passive” stretch and homogeneous cardiomyocyte alignment. The other possible scenario is that cardiomyocytes “actively” change their local cell-to-cell contact state to compensate contractility loss, which in turn leads to homogeneous alignment. In 3D histology, we have never seen less homogeneous alignment on endocardium where the stretching force seems to be weak compared with on epicardium. We also found that cultured cardiomyocytes on top of well-aligned platforms showed greater contractility by squeezing forces into constrained regions. These findings suggest that homogeneous cardiomyocyte alignment is rather due to “active” rearrangement of the heart to produce greater contractility, termed “alignment remodelling”, although our evidence is not strong enough to conclude the causal relationship. Then the next question is whether homogeneous alignment is beneficial for global cardiac function. Our in vitro results suggested that homogeneous alignment remodelling in DCM is beneficial at least in 2D. However, it can be detrimental in 3D because altered fibre orientation in DCM has been reported to decrease ejection fraction by disrupting torsion-ejection coupling^25^. Taken together, cardiomyocytes of DCM might rearrange their alignment locally towards homogeneous (“adaptive alignment remodelling”), but lose myocyte twist and global coordination as a trade-off (“maladaptive alignment remodelling”). Further in vivo perturbation of the alignment remodelling would be needed to determine this though.

From a clinical perspective, understanding molecular mechanisms of homogeneous alignment remodelling is important. Dysregulation of cell adhesion has been reported in the patients with DCM^26^. Also, protein expression levels of connexin43 and α-actinin, focal loss of desmosome integrity, cell membrane dissociation, and sarcomere and I-band elongation were observed in DCM mice that simultaneously exhibited loss of myocyte torsion^18^. Since molecules involved in these phenotypes might play a crucial role in regulating cardiomyocyte array^27–30^, cardiomyocyte-specific deletion of those genes and our 3D alignment evaluation method with intravital heart imaging would be helpful to investigate the mechanism underlying homogeneous array in DCM. Here we have established a novel method to evaluate cardiomyocyte array in 3D and demonstrated homogeneous alignment remodelling in DCM mice. Our findings will provide new insights into understanding the mechanism of heart failure.

## Methods

### Animal model

We used global double- fluorescent *Rosa26*^*mT/mG*^ reporter knock-in mice^17^ (mT/mG mice) purchased from Jackson Laboratory(stock#007576, stock name: Gt(ROSA)26Sor^tm4(ACTB-tdTomato,-EGFP)Luo^/J), and homogeneous knock-in mice with deletion mutation K210 in cardiac troponin T gene as DCM model mice^22^ (*TNNT*^ΔK210/ΔK210^ mice), kindly provided from Dr. Morimoto. We used homozygous mT/mG mice as control mice, double-homozygous mT/mG; *TNNT*^ΔK210/ΔK210^ mice as DCM model mice.

### Two-photon intravital imaging of beating hearts

Observation was performed according to our previous report ^10,16^. Briefly, mice (8-week old, male, n = 4 for each genetic background) were anesthetized by inhalation of isoflurane (5% for induction and 3% for maintenance) put under mechanical ventilation (0.7 mL tidal volume 150 times/min) (Shinano, Tokyo, Japan; Cat.No. SN-480-7) via an endotracheal intubation by 22-gauge tube. After anesthetizing, the forefeet and tube were fixed at the plate, and the anterior chest wall was cut off carefully with bipolar scissors (Force FX-CS, E4051CT; Valleylab, Denver, USA). The heart was exposed and fixed by gentle suction with handmade stabilizer. Then the heart was adjusted for observation with carefully avoiding bleeding (Fig. 1a).

The intravital microscope system was composed of a two-photon microscope (A1-MP; Nikon, Tokyo, Japan) with a laser (Chameleon Vision II Ti:Sapphire; Coherent, Santa Clara, CA, USA) tuned to 800–880 nm and an upright microscope equipped with a 25 × water immersion objective lens (CFI Apo 25 × W MP; Nikon). The mice on the plat were placed on a two-axis translation stage under the objective lens in a temperature-controlled dark box. The field of view was adjusted through Binoscope. After adjusting, the black-out curtain was closed and the light pathway was switched from the Binoscope to the front, and live imaging was initiated. Fluorescent signals were detected through band-pass emission filters at 492 nm, 525/50 nm, and 575/25 nm.

Three-color images (512 × 512 pixels) were acquired at 1.875 frames/s. For three-dimensional videos, sequential image stacks were acquired about 100 μm from the surface at 1 μm (Fig. 1b). After acquiring images, reconstruction into 3D image were performed (Fig. 1c). Raw data were processed with NIS-Elements software (NIKON).

### Immunofluorescence staining

Hearts were perfused with 4% (W/v) PFA in PBS after observation with two-photon microscopy. After perfusion, hearts were excised and immediately embedded in Tissue-Tek OCT cryo-embedding compound (Miles Laboratories). Cryostat sections along the long axis at 5 μm were fixed with 4% (W/v) PFA in PBS and incubated with primary antibody (anti-Troponin I antibody; ab56357, Abcam, 1:200) over night after blocking with 3% bovine serum albumin. After washing with PBS, samples were stained with appropriate secondary antibody (anti-goat IgG- Alexa 488 1:200) for 1 h. The nuclei of the cells were counterstained with TO-PRO-3 iodide 642/661 (Molecular Probes, 1:5000), respectively. Images of samples were acquired using LSM 700 confocal microscope (Carl Zeiss).

### Image analysis of angle distribution

On each 2D image, the angles of cardiomyocytes from a vertical line were drawn manually using ImageJ software (Fig. 1d). Then their distribution was plotted by R software (Fig. 1f). We will describe the details below.

### Heart tissue clearing and Image acquisition

Tissue clearing of the heart was performed in accordance with the CUBIC-perfusion protocol reported by Tainaka et al^31,32^. The CUBIC-1 reagent was prepared by mixing 25% urea (Wako Pre Chemical Industries, Osaka, Japan), 25% N,N,N′,N′-tetrakis (2-hydroxypropyl) ethylenediamine (Tokyo Chemical Industry Co., Ltd, Tokyo, Japan), and 15% Triton X-100 (Nacalai Tesque, Kyoto, Japan) in deionized water. The CUBIC-2 reagent was prepared by mixing 50% sucrose, 25% urea, 10% 2,2′,2″-nitrilotriethanol (Wako Pre Chemical Industries, Osaka, Japan), and 0.1% (v/v) Triton X-100 in deionized water. Both the CUBIC reagents were prepared and degassed before use.

For transcardial perfusion, a 21-gauge needle was inserted into the left ventricle through the apex. Mice were transcardially perfused with 10 mL of cold phosphate-buffered saline (PBS) containing 10 U/mL of heparin to remove the blood, 150 mL of cold 4% (w/v) PFA in PBS, 20 mL of PBS to wash out PFA, and 20 mL of 50% (v/v) CUBIC-1 reagent (1:1 mixture of PBS: CUBIC-1). Hearts were excised and continuously immersed in 30 mL of CUBIC-1 regent at 37 ℃ for 2 weeks with gentle shaking. The reagent was exchanged every day in the first week and every other day in the second week. After treatment with CUBIC-1 reagent, the hearts were washed with PBS three times for 30 min each at room temperature with gentle shaking, then immersed in 20% (w/v) sucrose in PBS at room temperature. On the next day, the samples were washed with PBS three times for 30 min each, and immersed in CUBIC-2 reagent with gentle shaking at 37 ℃ overnight. The next day, the CUBIC-2 reagent was changed and the samples were further incubated for several days.

Images of CUBIC-cleared hearts were acquired using the light-sheet microscope Lightsheet Z.1 (Carl Zeiss, Jena, Germany) equipped with a 5 × objective lens (EC Plan-Neofluar 5×, numerical aperture (NA) = 0.16, working distance (WD) = 18.5 mm) at 561 nm excitation. Fluorescent signals were detected through band-pass emission filters at 575/615 nm. Heart samples were immersed in CUBIC-2 reagent during image acquisition and sequential image stacks were obtained about 800 μm from the surface at 10 μm.

### Analysis of angle distribution on 3D histology

Images were subjected to a combined high and low pass filter and binarized, facilitating cell segmentation through the watershed algorithm. The distribution of angles cells make with their neighbours was determined through the MATLAB image processing toolbox (Mathworks,Natick, MA, USA). Cells within a defined arbitrary radius from the target cell were treated as neighbours, and together, defined a cells neighbourhood. The standard deviation of the angle distribution in a neighbourhood was used to infer local alignment heterogeneity.

### Cell culture

Hearts were excised from new-born (0-day-old) Wister rats and neonatal cardiomyocytes (NRCMs) were isolated using Neonatal Cardiomyocyte Isolation System (Worthington Biochemical Corp.) according to the manufactural instruction. NRCMs were sowed into seeded on 0.1% gelatine pre-coated linearly aligned nanofiber substrate (NanoAligned, 0802; Funakoshi, Tokyo, Japan) and randomly aligned nanofiber substrate (NanoECM, 0801; Funakoshi) and either at the density of 1.5 × 10^5^ cells/well. NRCMs were cultured in M-199 (12350-039; Gibco) supplemented with 10% fetal bovine serum (S1820-500; biowest) and 1% Penicillin–Streptomycin for 7 days.

### Motion analysis of spontaneously beating NRCMs

Free labelling motion analysis of NRCMs was performed using the cell motion imaging system (SI8000C, Cardio Model, Sony, Tokyo, Japan), as previously reported^33^. Maintaining spontaneously beating NRCMs at 37 °C in a 5% CO_2_, images were captured using a 10 × objective at a 150 frames/s for 10 s. The data were analysed with a SI8000C analyser software (Sony).

### Statistical analysis

All values are presented as mean ± SD. Statistical significance was analysed by unpaired two-tailed Student’s t-test. Significant difference was defined as *P* < 0.05. All data were analysed using the R software and hierarchical clustering was performed by the Morpheus software commercially provided by Broad Institute.

### Ethics statement

All methods were carried out in accordance with relevant guidelines and regulations. Animal experiments were approved by the Institutional Animal Care and Use Committee of Osaka University (27-021 and 27-032) and performed in conformity with ARRIVE guidelines.

## Supplementary Information

Supplementary Video 1.

Supplementary Video 2.

Supplementary Video 3.

Supplementary Video 4.

Supplementary Video 5.

Supplementary Information.
