# Supplementary material for: Homogeneous 2D and 3D alignment of cardiomyocyte in dilated cardiomyopathy revealed by intravital heart imaging
Source: Sci Rep. 2021 Jul 19;11:14698. doi: 10.1038/s41598-021-94100-z (PMC8289833; doi:10.1038/s41598-021-94100-z)
Supplement: Supplementary file 6 — Supplementary Information. [file 41598_2021_94100_MOESM6_ESM.pdf]

Homogeneous 2D and 3D Alignment of Cardiomyocyte in Dilated Cardiomyopathy Revealed by Intravital Heart Imaging

Kiyoshi Masuyama[1#], MD; Tomoaki Higo[1,2#*], MD, PhD; Jong-Kook Lee[3], MD, PhD; Ryohei Matsuura[4], MD, PhD; Ian Jones[2]; Chris Bakal, PhD[2]; Shuichiro Higo[1,5], MD, PhD; Sachio Morimoto[6], PhD; Shigeru Miyagawa[4], MD, PhD; Yoshiki Sawa[4], MD, PhD; & Yasushi Sakata[1], MD, PhD

Short title: Homogeneous Alignment Remodelling in Dilated Cardiomyopathy

[1]Department of Cardiovascular Medicine, Osaka University Graduate School of Medicine, 2-2 Yamadaoka, Suita 565-0871, Japan

[2]The Institute of Cancer Research, London, Chester Beatty Laboratories, 237 Fulham Road, London SW3 6JB, London, UK

[3]Department of Cardiovascular Regenerative Medicine, Osaka University Graduate School of Medicine, 2-2 Yamadaoka, Suita 565-0871, Japan

[4]Department of Cardiovascular Surgery, Osaka University Graduate School of Medicine, 2-2 Yamadaoka, Suita 565-0871, Japan

[5]Department of Medical Therapeutics for Heart Failure, Osaka University Graduate School of Medicine, 2-2 Yamadaoka, Suita 565-0871, Japan

[6]Department of Health and Medical Care, International University of Health and Welfare, Okawa, Fukuoka, 831-8501, Japan

#These authors contributed equally to this work.

Correspondence to:

Tomoaki Higo, Department of Cardiovascular Medicine, Osaka University Graduate School of Medicine, 2-2 Yamadaoka, Suita 565-0871, Japan,

Fax +81-6-6879-3639

Tel +81-6-6879-3640

E-mail: tomohigo@cardiology.med.osaka-u.ac.jp

SUPPLEMENTAL MATERIAL

Supplemental Figure 1

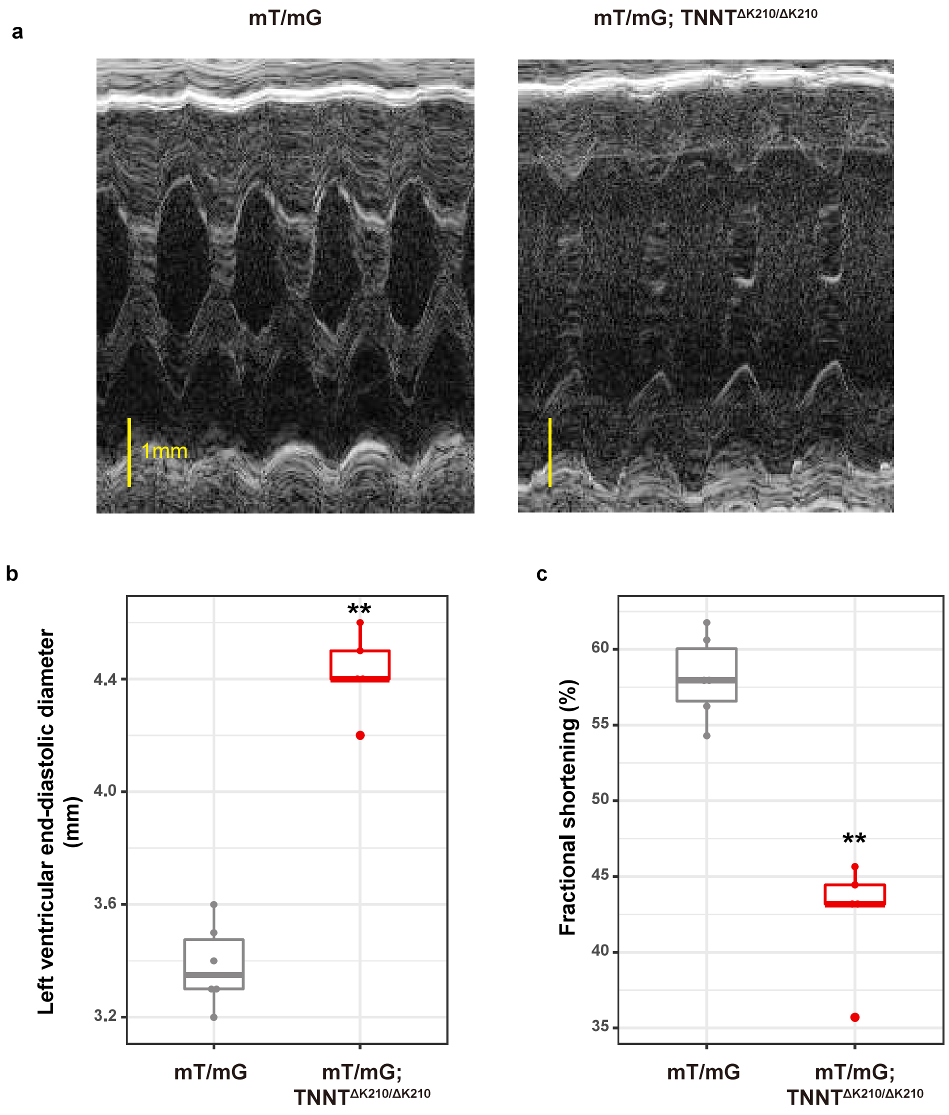

Supplemental Figure 2

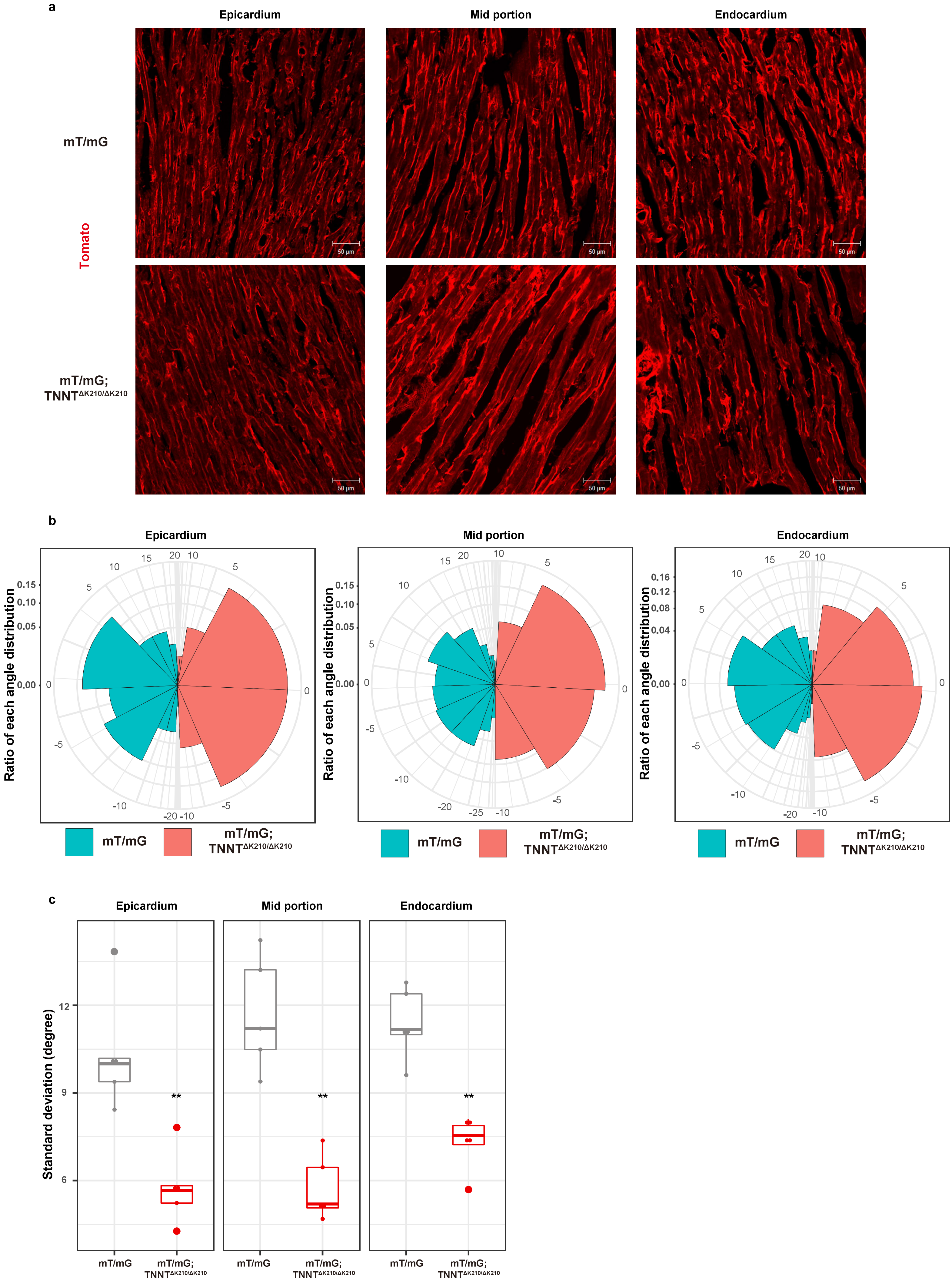

Supplementary Movie 1

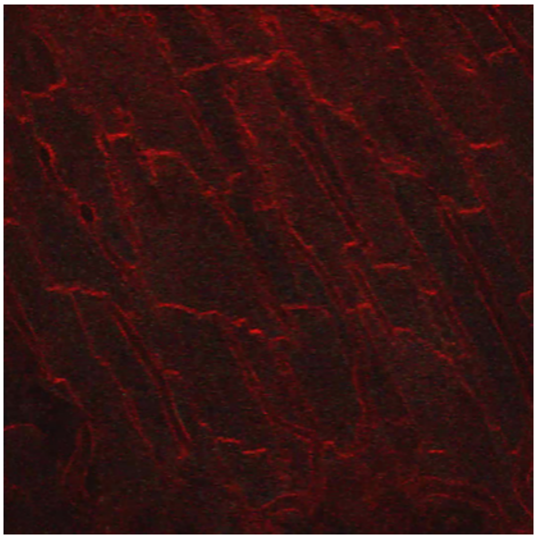

Supplementary Movie 2

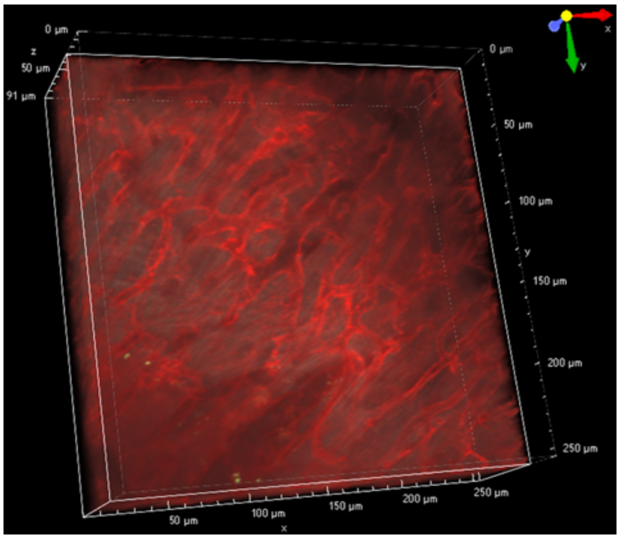

Supplementary Movie 3

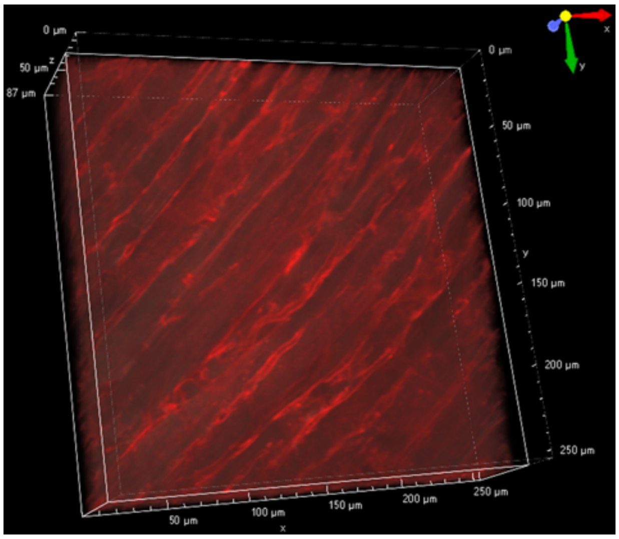

Supplementary Movie 4

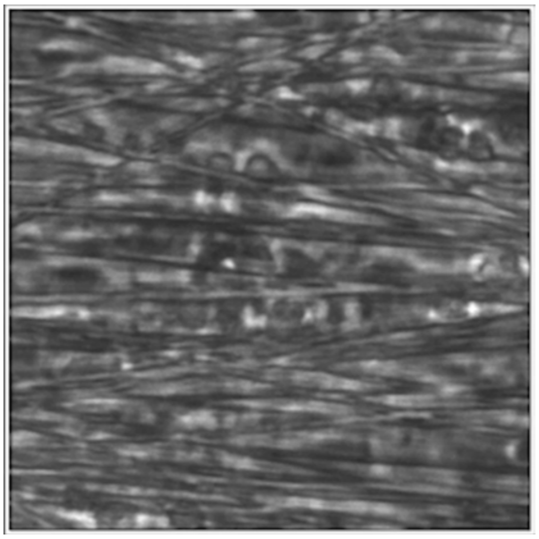

Supplementary Movie 5

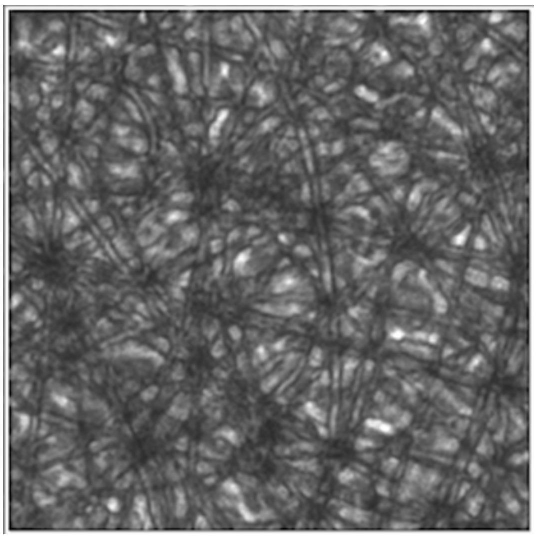

Supplementary Figure legend

Supplementary Figure 1.

a, Echocardiographic images of mT/mG mice and in mT/mG; *TNNT*$^{\Delta K210/\Delta K210}$ mice. Scale bar, 1mm. **b,c,** Left ventricular end-systolic diameter (**b**) and fractional shortening (**c**) were measured and statistically analysed (biological replicate=6, 5 respectively). Statistical significance was determined by unpaired two-tailed Student's t-test. **P<0.01 vs mT/mG mice. A box and a line above/below the box indicate SD and 95% confidence interval, respectively. All statistical analyses were performed with the R software and **Supplementary Figure 1b, c** were created using the R software.

Supplementary Figure 2.

a, Heart sections on epicardium, mid portion, and endocardium level of mT/mG mice and mT/mG; *TNNT*$^{\Delta K210/\Delta K210}$ mice were evaluated. Cardiomyocyte membranes were genetically labelled with a Tomato fluorescent reporter protein (red). Scale bar, 50µm. **b,** Angle distribution of cardiomyocytes on immunostained heart tissue sections of epicardium, mid portion, and endocarduim level were compared and visualized on a pie chart between mT/mG mice (light green) and mT/mG; *TNNT*$^{\Delta K210/\Delta K210}$ mice (pink). On each image, longitudinal lines of cardiomyocytes were manually drawn and angles from

a horizontal line was measured and calculated angles from median angle, ranging from -90 ° to 90 °. **c,** Representative data of standard deviation of all the angles on each image was compared between mT/mG mice (grey) and mT/mG; *TNNT*$^{\Delta K210/\Delta K210}$ mice (red) on epicardium, myocardium, endocardium. (5slices per section).***P*<0.01 vs mT/mG mice. All statistical analyses were performed with the R software and **Supplementary Figure 2b, c** were created using the R software.

Supplementary Movie 1

Beating hearts in mT/mG mice were observed with intravital heart imaging. The movie was acquired at 30 frames/s. Genetically labelled with a membrane Tomato fluorescent reporter protein enabled to detect cardiomyocyte borders.

Supplementary Movie 2 and 3

Representative 3D images of mT/mG mice (**Movie 2**) and mT/mG; *TNNT*$^{\Delta K210/\Delta K210}$ mice (**Movie 3**) reconstructed from approximately 100 consecutive 2D images captured by two photon microscopy at 1.875 frames/s.

Supplementary Movie 4 and 5

Representative movie of linearly (**Movie 4**) and randomly (**Movie 5**) aligned NRCMs in the 256 × 256 pixel-region of interest recorded by the SONY motion analyser at 150 frames/s.
